# Evolution of Nanotechnology in Delivering Drugs to Eyes, Skin and Wounds via Topical Route

**DOI:** 10.3390/ph13080167

**Published:** 2020-07-27

**Authors:** Pratheeksha Koppa Raghu, Kuldeep K. Bansal, Pradip Thakor, Valamla Bhavana, Jitender Madan, Jessica M. Rosenholm, Neelesh Kumar Mehra

**Affiliations:** 1 Pharmaceutical Nanotechnology Research Laboratory, Department of Pharmaceutics, National Institute of Pharmaceutical Education and Research, Hyderabad 500037, Telangana, India; pratheekshakr1995@gmail.com (P.K.R.); thakor.pradip291@gmail.com (P.T.); 23bhavana11@gmail.com (V.B.); jitenderpharmacy@gmail.com (J.M.); 2 Pharmaceutical Sciences Laboratory, Faculty of Science and Engineering, Åbo Akademi University, 20520 Turku, Finland; jessica.rosenholm@abo.fi

**Keywords:** nanomedicine, topical route, eyes, skin, nanotechnology, drug delivery

## Abstract

The topical route is the most preferred one for administering drugs to eyes, skin and wounds for reaching enhanced efficacy and to improve patient compliance. Topical administration of drugs via conventional dosage forms such as solutions, creams and so forth to the eyes is associated with very low bioavailability (less than 5%) and hence, we cannot rely on these for delivering drugs to eyes more efficiently. An intravitreal injection is another popular drug delivery regime but is associated with complications like intravitreal hemorrhage, retinal detachment, endophthalmitis, and cataracts. The skin has a complex structure that serves as numerous physiological barriers to the entry of exogenous substances. Drug localization is an important aspect of some dermal diseases and requires directed delivery of the active substance to the diseased cells, which is challenging with current approaches. Existing therapies used for wound healing are costly, and they involve long-lasting treatments with 70% chance of recurrence of ulcers. Nanotechnology is a novel and highly potential technology for designing formulations that would improve the efficiency of delivering drugs via the topical route. This review involves a discussion about how nanotechnology-driven drug delivery systems have evolved, and their potential in overcoming the natural barriers for delivering drugs to eyes, skin and wounds.

## 1. Introduction

The oral route is the most preferred route of drug delivery for management of several diseases. When the oral route is used for treating topical infections of skin and eyes, drugs delivered systemically might cause adverse effects due to non-specific drug exposure [1]. In contrast, application of conventional dosage forms locally is not able to provide sustained drug release, and may not be efficient in overcoming the anatomical barrier effect at the diseased site. However, the emergence of nanotechnology in the field of drug delivery has shown to be a promising tool in overcoming these difficulties. The advancement in nanotechnology has paved the way for topical drug delivery by providing possibilities for sustained drug release, enhanced localized effects by overcoming the natural barriers and reduction in toxicity [2]. Researchers have been continuously working on various nanotechnology-driven drug delivery systems such as dendrimers, micelles, solid lipid nanoparticles (SLNs), liposomes, nanostructured lipid carriers (NLC), nanoemulsions and nano-crystals. However, low loading capacity, toxicity, stability scale-up, batch-to-batch reproducibility and clinical performance are the major challenges in the development of nanotechnology-driven products.

The human eye is an anatomically complex structure, due to which delivering drugs to eyes remains a challenge [3]. The drugs applied onto the eye have to be either retained at the cornea and/or conjunctiva, or have to cross these barriers to reach the targeted ocular site. For treating diseases associated with the back of the eye, drugs have to diffuse through a gel-like, very dense matrix called the vitreous humor [4]. The topical route is the most preferred route for delivering drugs to eyes due to its non-invasive nature with minimal side effects. However, the conventional topical drug delivery systems have shown an ocular bioavailability of less than 5% due to blinking, rapid tear turnover and drainage in the eyes [5]. 

Skin is the first line of defense of the human body against the entry of exogenous chemicals and microorganisms with *stratum corneum* as a major barricade [6,7]. There could be two diffusional routes for a drug molecule to penetrate through the skin surface, i.e., transappendageal and transepidermal route. The transappendageal route includes transport through the sweat glands and hair follicles. The transepidermal route involves two main pathways: (i) transcellular—involving passage of drug molecules across the corneocytes and lipid matrix, (ii) intercellular—involving movement of drug molecules in between the corneocytes [8]. Several methods have been employed to improve the penetration of drugs through the skin, such as penetration enhancers (chemicals that interact with the skin components to promote drug flux) [9], prodrugs [10], iontophoresis [11], magnetophoresis [12], and nanotechnology-based drug delivery systems. Nevertheless, due to the unique characteristics and small size of nanotechnology-based drug delivery systems, these especially may offer efficient, site specific and controlled delivery of drugs via the topical route.

Age, obesity, smoking and chronic diseases such as diabetes, arterial and venous insufficiency are factors known to delay wound healing and enhance the risk of developing chronic wounds. These chronic wounds are a challenging clinical problem worldwide. It has been reported that the elderly population is more prone to chronic wounds. In the United States, 3% of the population who are above 65 years of age have open wounds. It is estimated that in 2020, the population of elderly American people would reach over 55 million, which indicates that chronic wounds would remain an increasingly persistent problem in this age group [13]. The wound healing process involves phases like inflammatory, proliferative and remodeling that occur sequentially. Chronic wounds fail to go through these sequential phases, resulting in delayed wound healing. Current therapies for wound healing such as tissue engineered skin substitutes are expensive, and a repetitive administration regime is required for the recovery of patients as well as there being high chances of ulcer recurrence [13]. Hence, there is a need to develop novel drug delivery systems for sustained drug release at the wound site, which are also capable of reducing the recurrence of ulcers. Statistics shows that the market for topical medicines was worth about USD 92.40 billion in 2016 and is expected to reach approximately USD 125.88 billion by 2021 [14]. Therefore, considering the advantages of nanotechnology, with this review we attempt to compile the recent advancements on topical nanoformulations for skin and eye diseases and wound healing. We first narrate the barriers involved in topical drug delivery and then shed light on the superiority of nano-based delivery systems in overcoming these barriers. 

## 2. Nanotechnology in Ocular Drug Delivery

### 2.1. Anatomical Structure of Eye

**Figure 1:**
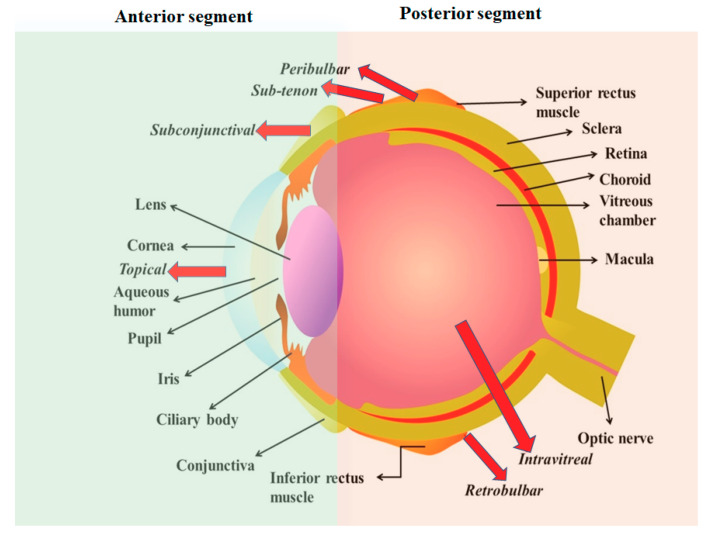
Anatomy of the human eye and routes of drug administration. Black arrows show different parts of human eye and red arrows show various routes of drug administration. Reproduced with permission from reference [5], copyright 2017 Elsevier B.V.

The detailed anatomical structure of the eye and its routes of administration are presented in Figure 1. The anatomical structure of the eye is composed of two parts, i.e., anterior segment and posterior segment [15,16,17,18,19]. Paracellular and transcellular are the two routes for penetration of drugs into the corneal epithelium. The penetration of lipophilic molecules predominantly involves transcellular mechanisms, however, hydrophilic molecules and ions involve the paracellular route [19].

### 2.2. Barriers for Topical Drug Delivery to Eyes 

The anatomical structure of the human eye is very complex, making ocular drug delivery a challenge for formulation scientists [20]. Numerous physiological barriers restrict the delivery of drugs into the anterior and posterior segment of the eyes. Depending upon the site of pathology, drugs have to be retained at the cornea or conjunctiva or may have to reach the inner structures of the eye. Drugs entering the conjunctiva usually undergo systemic absorption, which leaves the cornea as a sole route for delivering drugs to the inner parts of the eye. The cornea is made up of highly organized corneal epithelium, and has hydrophilic stroma that make the transport of drugs through the cornea difficult [4]. A gel-like structure known as vitreous humor, poses difficulty for the drugs to pass through, thus hindering the attainment of the optimum amount of drugs to the posterior segment [21]. Therefore, there is a need to design drug delivery systems that aid drugs in overcoming these barriers in order to reach the target site. 

### 2.3. Nanotechnology in Overcoming the Barriers of Topical Delivery to the Eye 

Delivery of drugs into ocular tissues is one of the most interesting and challenging aspects to formulators, due to the complex structure and physiology of the eye. In the last few decades, nanotechnology has shown promising results in the management of ocular diseases through realizing controlled drug release, minimizing eye irritation, and enhancing drug bioavailability as well as ocular tissue compatibility. Numerous nanosystems with specific designs are available to deliver their payloads into both the anterior and posterior segments of the eye. Depending on the particle charge, surface characteristics and relative hydrophobicity, nanoparticles can be designed to overcome the retinal barriers, including the blood–retinal barrier. Additionally, nanosystems can provide protection to the drug from degradation and are able to release the encapsulated drug in a controlled and sustained fashion [15,16].

Some ophthalmic products have the requirement of a repetitive dosing, such as drugs used for chronic cytomegalovirus retinitis (CMV). Therefore, long-term therapy is necessary for the management of such diseases, which may lead to cataract development, retinal detachment and endophthalmitis [5]. However, nanoparticles prepared using natural polymers, e.g., albumin, demonstrate promising therapeutic outcomes for such disease conditions [22]. Upon eye instillation, they did not induce any inflammatory reactions in the retinal tissue nor disturb the surrounding ocular tissues. Nanotechnology-mediated ophthalmic products are spurring enormous interest in retinal drug delivery, because retinal neovascularization and choroidal neovascularization have a similar microenvironment as solid tumors, where enhanced permeation and retention (EPR) effect is also playing a role for choroidal neovascularization and drug targeting using nanoparticles [23]. The EPR effect is a postulation by which molecules of certain sizes (typically liposomes, nanoparticles, and macromolecular drugs) tend to accumulate in tumor tissues much more than they do in normal tissues [24]. Different nanosized drug delivery systems applied to various ocular diseases are discussed below.

### 2.4. Nanotechnology-Driven Drug Carriers for Topical Delivery to Eyes

Various nanotechnology-driven drug carriers are summarized in Table 1 and are discussed in the following.

#### 2.4.1. Liposomes

Liposomes are made up of natural biocompatible phospholipids that delivers drugs in a sustained-release manner to the eyes [20]. The structure of liposomal vesicles allows them to encapsulate hydrophilic drugs in the aqueous core, while hydrophobic or amphiphilic drugs can be embedded in the lipid layers [20,25]. The liposomal vesicles vary in size from 10 to 1000 nm [26]. The drug in the liposomes is delivered to the eyes via four mechanisms:

(1) Adsorption: Adsorption of liposomes onto the cell membrane is the first step in delivering the drugs from the liposomes. In the presence of cell surface proteins, liposomes become leaky and release their contents near the cell membrane. This results in a high drug concentration in the vicinity of the cell membrane, and promotes the cellular uptake of drugs by passive diffusion [26].
(2) Endocytosis: After adsorption and cellular uptake, the liposome reaches into the endosomes, and is then transported to the lysosomes through endosomes. Later, the enzymes in the lysosomes degrade the lipids and the entrapped drug will be released into the cytoplasm [26].
(3) Fusion: Fusion of the lipid bilayer of liposomes with lipoidal cell membrane by intermixing and lateral diffusion of lipids results in direct delivery of liposomal contents into the cytoplasm [26].
(4) Lipid exchange: Due to the similarity in the lipids present in the liposomal membrane and the phospholipids present in the cell membrane, lipid transfer proteins in the cell membrane recognize liposomes and therefore cause lipid exchange. As a result of this, the liposomal membrane gets destabilized and the drug gets released [26].

Liposomes are said to enhance the permeability of hydrophilic, lipophilic and amphiphilic drugs through the cornea. There are many factors influencing drug delivery efficiency through liposomes. The surface charge of liposomes is one, and cationic liposomes have been shown to have a prolonged residence time in the cornea since they strongly interact with the cornea due to its negative charge [26]. Multi-lamellar vesicles are shown to be retained for a longer time than the small unilamellar vesicles, which may be attributed to the restricted drainage from the inner canthal region [26].

Liposomes have evolved from conventional to the new generation, in order to enhance safety and efficacy of this carrier system. Conventional liposomes generally have an outer lipid layer and an inner aqueous core, whereas the new generation liposomes—also known as stealth liposomes—are coated with polyethylene glycol (PEG), and protect them from engulfment by phagocytic cells [27]. Another class of liposomes are immunoliposomes, which have been developed for targeted delivery by conjugating antibodies at the surface that recognizes specific proteins on the target cells [28,29,30]. Cationic liposomes consist of positively charged lipids that interact efficiently with the negatively charged DNA and thereby condense the DNA into a more compact structure; hence, they are mostly used for delivering genes into eyes into various diseases such as glaucoma, epithelial retinal diseases, cytomegalovirus retinitis, etc. These lipid complexes provide protection to the entrapped genetic material and enhance its intracellular delivery. In some cases, the cationic liposome also contains helper lipids such as di-oleoyl phosphatidylethanolamine, which stabilize the liposome complex. Among the new generation liposomes, cationic liposomal formulations are the youngest members and hold a promising future, particularly for delivering genes to the eyes [31,32]. The zeta potential of cationic liposomes must be between 10 to 30 mV [33].

Kawakami and co-workers prepared cationic liposomes with plasmid DNA and investigated the in vivo transfection efficiency in rabbits after intravitreal injection (100 µL). Plasmid DNA alone does not show significant transfection efficiency from 40 to 85 mg, however, pDNA complexed with *N*-[1-(2,3-dioleyloxy)propyl]-*N*,*N*,*N*-trimethylammonium chloride (DOTMA)/Cholesterol (chol) liposomes significantly increased the pDNA ranging from 40 to 85 µg (*p* < 0.05). It was difficult to prepare the complex of pDNA with the DOTMA/Chol liposomal formulation having more than 60 µg pDNA. These liposomes (pDNA; 85 mg/DOTMA/Chol) showed high transfection efficiency and luciferase activity in the cornea, aqueous humor, iris-ciliary body, lens, vitreous body and retina for 3 days, but at the same time showed high cytotoxicity and aggregation behavior with the retina, which decreases visual activity and causes blindness. Therefore, an anionic coating of natural polysaccharides or synthetic polymer is applied to reduce cytotoxicity as well as aggregation behaviors [34].

Liu and coworkers formulated PEGylated liposome-protamine-hyaluronic acid nanoparticles (PEG-LPH-NP) loaded with siRNA (PEG-LPH-NP-S) for the treatment of choroidal neovascularization (CNV) on a laser-induced rat model. siRNA directed against vascular endothelial growth factor (VEGF) or VEGF receptors in CNV animal models have shown promising results. The PEG-LPH-NP-S were 132 nm in size with 20 mV cationic zeta potential, and depicted >95% encapsulation efficiency. It was found that PEG-LPH-NP could efficiently protect the siRNA load, facilitate the intracellular delivery of siRNA and the expression inhibition of VEGFR1. Apoptosis was detected by the terminal deoxynucleotidyl transferase-mediated dUTP-biotin nick ending-labeling method (TUNEL). The TUNEL testing and morphologic observation showed low toxicity on the rat retina, and thus, these lioposomes were considered as a safe nanoformulation in the treatment of choroidal neovascularization [35].

In another study, the delivery efficiency of diclofenac-loaded liposomes to the retina was compared with conventional diclofenac ophthalmic solution on rabbit eyes. It was reported that the liposomes modified with PVA or polyvinyl alcohol derivatives bearing a hydrophobic anchor (C16H33 S) at the terminal of the molecule (PVA-R) improved the physical stability of diclofenac-loaded liposomes. After administration to eye, PVA-R-liposomes took the non-corneal pathway to deliver diclofenac efficiently to the retina. These liposomes also displayed better physical stability and less aggregation [36].

It has been suggested that bioadhesive and biodegradable polymers can be used to prolong the residence time of liposomal formulations. Mehanna et al. demonstrated that chitosan-coated liposomes encapsulated with antibiotic ciprofloxacin HCl improved the ocular permeation of the drug. This formulation inhibited the growth of *P. aeruginosa* in rabbit eyes for 24 h when compared to the marketed preparation [37]. Fernandez de Sá et al. designed voriconazole-loaded liposomes composed of soy phosphatidylcholine for fungal keratitis, which demonstrated the delivery of reasonable amounts of drug to the cornea [38]. 

#### 2.4.2. Niosomes

Niosomes are vesicles similar to liposomes, but unlike liposomes they are composed of non-ionic surfactants. Similarly to liposomes, they can encapsulate both hydrophilic and hydrophobic as well amphiphilic compounds. Niosomes were investigated to deliver the drug timolol maleate, widely prescribed for glaucoma. It was found that 80% of the drug was lost either because of rapid blinking of eyes or due to drainage into the nasolacrimal duct while using as a conventional eye drop. In addition, drug drainage into the nasolacrimal duct results in systemic side effects such as heart disease or asthma. In contrast, niosomes loaded with timolol maleate demonstrate better retention and penetration of the drug through the cornea and, at the same time, reduced side effects. The size of prepared niosomal formulation was found be in the range of 2 to 3 µm with entrapment efficiency (EE) of 24.3% *w*/*v*. The prepared niosomal formulation provided prolonged and controlled release as compared to the marketed solution (Timolet^®^ GFS, Sun Pharmaceuticals India Limited, Vadodara, India. A higher concentration of the drug was found across the corneal barrier of the eye in niosomal formulation. These results indicated that the niosomal formulation has a higher permeation capacity as compared to marketed niosomal formulation. The prepared niosomal formulation used less than 50% of timolol meleate which produce fewer side effects [39]. 

Abdelbary and co-workers prepared an ophthalmic niosomal formulation for controlled delivery of gentamycin. Niosomal formulations were prepared using various surfactants (Tween 60, Tween 80 or Brij 35), in the presence of cholesterol and a negative charge inducer dicetyl phosphate (DCP) in different molar ratios using a thin film hydration technique. An in vitro study suggested that niosomal formulation exhibited high drug retention in vesicles and showed sustained drug release as compared to the free drug solution. Prepared niosomal formulations were tested for ocular irritation and it was found that the prepared niosomal formulation did not produce irritation in albino rats [40].

#### 2.4.3. Nanoparticles

The first prototype of polymeric nanoparticles used for topical ocular delivery was made from poly-(alkyl cyanoacrylates) (PACA). Polymers that are commonly used for preparing nanoparticles are poly-ε-caprolactone (PCL), chitosan, poly-(lactic-co-glycolic acid) (PLGA), carbopol, gelatin, Eudragit^®^ (RS100 and RL100), hyaluronic acid and so forth [4]. Gold, silver, silica and cerium oxide nanoparticles are also used as drug carriers for ocular delivery [15]. Nanocapsules and nanospheres are the two main types of nanoparticles with distinct structures. As stated in the introduction, nanoparticles have many advantages over the conventional drug delivery systems that can be exploited in ocular drug delivery including better penetration through biological barriers, potential for targeted delivery and controlled drug release.

Similarly to cationic liposomes, nanoparticles with positive surface charge demonstrate better corneal retention and permeability [41]. Vasconcelos et al. employed a PLGA-b-PEG copolymer to conjugate a cell penetrating positively charged peptide for improving ocular penetration of the prepared nanoparticles loaded with flurbiprofen. Nanoparticles fabricated using the abovementioned conjugate demonstrated sustained release (for up to 8 h) and high payload of drug as well as depicting low ocular toxicity [42].

Chitosan-based nanoparticles loaded with ketorolac tromethamine were studied by Fathalla et al. for their enhanced delivery to the eyes. The size of the NPs was affected bychitosan/tripolyphosphate (CS/TPP) ratio where the diameter of the NPs ranged from 108.0 ± 2.4 nm to 257.2 ± 18.6 nm. The zeta potential was found to be in the range of 22.9 to 27.8 mV. The positive charge of NPs of different formulation was obtained due to the cationic nature of chitosan. The in vitro release study demonstrated a sustained release of the drug over a period of 6 h from the nanoparticles compared to a conventional solution, which was released within 3 h. The ex vivo studies conducted using porcine eye balls confirmed the ability of the prepared nanoparticles to retain the drug on the corneal surface for a longer time compared to the ketorolac tromethamine solution [43].

Naringenin is being used for treating age-related macular degeneration (AMD), however, its use is limited due to the poor aqueous solubility. Hence, nanoparticular carrier systems have been examined for its ocular drug delivery. Naringenin (Nag)-loaded sulfobutyl ether-β-cyclodextrin/chitosan nanoparticles (Nag CD/CS-NPs) were formulated using an ionic gelation method of chitosan with butyl ether-β-cyclodextrin (SBE-β-CD). The Nag CD/CS-NPs with positive zeta potential (~22 mV) and size around 450 nm released the drug in a sustained manner. In vitro release studies were evaluated in simulated tear fluid (STF) using a dialysis tube with a molecular weight cutoff of 8000–14,000. After 5 h, Nag-CD gave 57% drug release whereas Nag CD/CS-NPs gave only 38% drug release. These results indicated that the coated nanoparticle complex provided a sustained release of drug as compared to the only cyclodextrin complex. The in vivo study was carried out on New Zealand white rabbits. The in vivo study showed that Nag CD/CS-NPs had a much higher ophthalmic bioavailability and prolonged residence time, which significantly increased Nag bioavailability in the aqueous humor compared to the suspension formulation and, consequently, reduced the frequency of administration [44].

#### 2.4.4. Polymeric Micelles

Polymeric micelles are also investigated for delivering drugs to both the anterior and posterior segments of eye, due to their ability to increase the solubility of hydrophobic drugs and to enhance drug absorption across biological barriers including the ones in the eye. Their ability to bind with cellular barriers and improve penetration of drugs through the cornea may be attributed to their similarity in size range to the membrane proteins, scleral pores (cornea), and other biomolecules. Polymeric micelles are a potential carrier to deliver hydrophobic drugs and proteins through biological membranes, to increase the chemical stability of unstable compounds, and to control the release of drugs [15,45].

Luschmann et al. prepared an in-situ forming nanosuspension made of liquid poly(ethylene glycols) (PEG) and a simple micellar solution containing nonionic surfactants that increase cyclosporine A solubility. The in vivo experiments suggested that the micellar solution is suitable for delivering drugs to the anterior segment of eye. The in-situ nanosuspension and the micellar solution deliver high levels of drug to eye, i.e., 1683 ± 430 ng CsA/g cornea and 826 ± 163 ng CsA/g cornea, respectively. Both formulations exceeded the drug tissue levels reported for Restasis^®^ (350 ng CsA/g cornea) and cationic emulsions (750 ngCsA/g cornea). Despite the neutral charge of micelles, concentrations of drug at tissues levels were slightly higher as compared to cationic emulsion [46].

In another similar study, cyclosporine A micelles were prepared using polyvinyl caprolactam-polyvinyl acetate-polyethylene glycol graft copolymer (PVCL-PVA-PEG), also known as Soluplus^®^. The Soluplus micelles were able to deliver an adequate amount of Cyclosporine A into the cornea for the treatment of immune-mediated corneal disease. The developed formulation also displayed a good ocular tolerance in rabbit eyes [47].

Antioxidant α-lipoic acid (ALA) is used for treating diabetic keratopathy and retinopathy. Anterior eye segment structures are usually damaged in corneal neuropathy and keratopathy. Micelles prepared using Soluplus were investigated for their ability to solubilize ALA and the prepared formulation was compared with a marketed eye drop that was obtained using conventional micelles of sodium dioctylsulfo succinate surfactant. Prepared Soluplus-based nanoformulation was of around 70–80 nm in size and increased the solubility of ALA by around 10 times compared to the commercial eye drop. These micelles enhanced the flux of α-lipoic acid through cornea and were found to be less irritant [48].

As an example of targeted delivery to the eyes, Boddu and co-workers developed doxorubicin entrapped local in-situ gel utilizing folic acid-PEG-PLGA copolymer micelles for ligand-mediated targeted delivery to retinoblastoma cells via intravitreal administration. After intravitreal administration, DOX micelles in PLGA-PEG-PLGA gel gave sustained release for up to two weeks. The uptake of DOX in micellar solution was four times greater than plain DOX solution [49].

#### 2.4.5. Dendrimers

Dendrimers are “tree-like” nanostructured polymers that have been interesting in terms of ocular drug delivery. They are attractive systems for drug delivery due to their nanosize range, ability to display multiple surface groups that allows for targeting, and easy preparation and functionalization. Dendrimers demonstrate promising properties in topical delivery of drugs to the eyes. Holden et al. encapsulated the two anti-glaucoma drugs brimonidine and timolol maleate into dendrimer hydrogel (DH) made up of polyamidoamine (PAMAM) dendrimers tethered with multiple PEG chains and photoreactive acrylate groups. They investigated the intracellular uptake of these two encapsulated drugs on human corneal epithelial (HCE) cells and the transport across the bovine corneal endothelium. The prepared DH formulation showed mucoadhesive properties towards mucin particles and were non-toxic to human corneal epithelial cells, and depicted higher HCE uptake and significantly increased bovine corneal transport for both anti-glaucoma drugs, as compared to the conventional eye drop [50].

Yavuz et al. investigated anionic and cationic PAMAM dendrimer complexes for delivering dexamethasone to the posterior segment of the eye (retina) for the treatment of diabetic retinopathy. An ex vivo transport study using rabbit cornea and sclera-choroid retina pigment epithelium (SCRPE) tissues demonstrated that anionic dendrimers delivered higher drug concentrations at the corneal and scleral tissues compared to the free drug solution and cationic dendrimers. From this finding, it was concluded that dexamethasone-PAMAM complex formulations enhanced ocular bioavailability of dexamethasone via topical route [51].

Lancina et al. designed fast dissolving dendrimer-based nanofibers (DNF) as a topical delivery vehicle for brimonidine tartrate for glaucoma, where DNF showed better efficacy than pristine brimonidine tartrate solution [52]. The water-soluble anionic, cationic carbosilane dendrimers (generation 1–3) was explored in eye drop solution as muco-adhesive polymer containing acetazolamide (ACZ) in normotensive rabbits for improved hypotensive effect of ACZ. Authors investigated both the cationic and anionic modified carbosilane dendrimers using human ocular epithelial cell lines and rabbit eye for in vitro and in vivo tolerance studies, respectively. This study indicates that both carbosilane dendrimers are well-tolerated in concentration between 5 and 10 µM. An eye drop formulation comprising G3 carbosilane dendrimers (5 μM) and ACZ (0.07%) induced a rapid (onset time 1 h) and extended (up to 7 h) hypotensive effect which led to a significant increment in the therapeutic efficacy with area under the plasma drug concentration (AUC0) (8 h), along with the maximal intraocular pressure reduction. These research finding suggest that the dendrimer-mediated topical eye formulation is safe and can be commercialized for treatment of eye diseases [53].

#### 2.4.6. Nano-Implants

Implantable technology is not new and has been in use for many decades; however, the incorporation of micro- and nanotechnologies in implants enables the development of safe and more efficient drug delivery devices. Advancement in implants creates a huge impact on patient comfort and compliance, while treating chronic conditions by delivering drugs in a sustained manner to the posterior segment of eyes (posterior segment eye disease). These technologies are scalable technologies and offer localized drug delivery for a prolonged duration.

Some nanocontrolled release systems that are implanted into the eyes to achieve drug delivery are already in clinical practices (e.g., Vitrasert^®^, Retrisert^®^, Surodex™ and Ozurdex^®^). Biodegradable polymers including both synthetic (most commonly used for ocular implants), e.g., polylactic acid (PLA), polyglycolic acid (PGA), PLGA, PCL, polyanhydrides (PA) and polyortho-esters (POE), and natural polymers such as collagen, gelatin and chitosan have been utilized to fabricate nano-implants [54].

Vitrasert^®^ is an example of intravitreal implant technology useful for cytomegalovirus retinitis and HIV infections comprising of ganciclovir formulated using semipermeable poly vinyl alcohol (PVA), which allows for drug release and an impermeable ethyl vinyl acetate polymer (EVA). The implant tablet contains 4.5 mg of drug and 0.015 mg magnesium stearate, which release from all sides of the tablet except the discontinuous EVA layer. The discontinuity in the EVA coating creates a diffusion port for the diffusion of ganciclovir from the implant into the eye. This technology releases active medicaments for 5–8 months and is devoid of systemic toxicity and thus cuts the costs of treatment. In the clinical studies, median time to disease progression of 6–8 months was observed in patients treated with a Vitrasert implant. The shelf life of the implant is 24 months and it is stored at a temperature between 15 and 25 °C [55,56]. Retisert^®^ is a United State of Food & Drug Administration (USFDA)-approved steroid implant manufactured by Bausch & Lomb, Rochester, NY, USA by incorporation of 0.59 mg of fluocinolone acetonide (FA) for controlled delivery into vitreous cavity (approximately 0.6 µg/day). It can release fluocinolone slowly for up to 3 years. In the long term, drug retention after release from the Retisert^®^ effectively suppresses the inflammation, reduces the recurrence of uveitis and improves visual acuity in 80% of patients [56,57].

Surodex™ and Ozurdex^®^ are other examples of biodegradable implants that are widely used in clinical practice for intraocular delivery and sustained release of dexamethasone. Surodex™ is implanted into the atria to treat the post-operative inflammation of cataract patients and comprises 60 µg of dexamethasone into the PLGA hydroxypropyl methylcellulose polymer matrix. Ozurdex^®^ is an intravitreal implant containing 0.7 mg of dexamethasone in PLGA polymer, which is capable of releasing the drug at a slow rate for up to 6 months. It is preloaded into a single-use drug delivery system (DDS) device for direct injection into the intravitreal cavity, administrated with the assistance of a healthcare expert. Clinical trial data suggest its potency in reducing vision loss. Data also suggest the improvement in vision acuity in eyes with macular edema associated with branch retinal vein occlusion (BRVO) or central retinal vein occlusion [54,56].

## 3. Topical Drug Delivery to Skin

### 3.1. Anatomy of Skin

**Figure 2:**
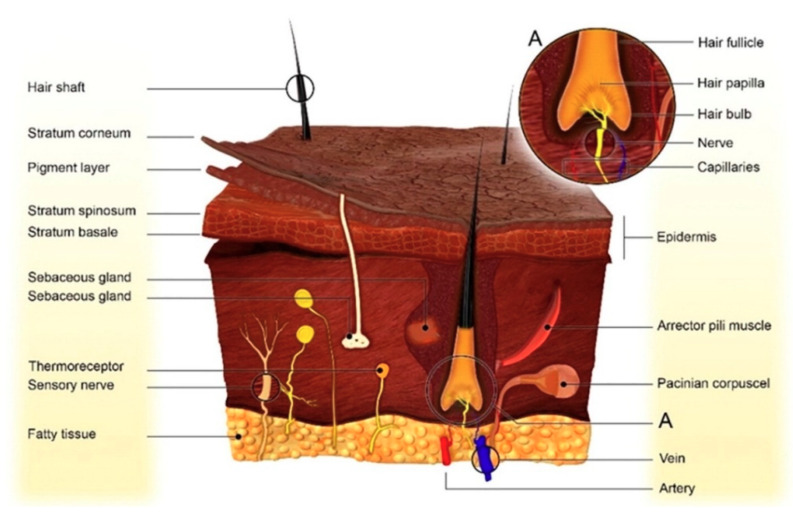
Anatomical structure of skin (reproduced with permission from [67], copyright 2019 Elsevier B.V.).

Skin has a complex structure that serves as a physical barrier to the entry of exogenous substances. Its detailed anatomical structure is presented in Figure 2. Skin is composed of three distinct layers, i.e., epidermis, dermis and hypodermis [66]. The presence of numerous physiological barriers to the different layers of skin poses challenges to the pharmaceutical researchers for effective drug transport through the skin.

### 3.2. Barriers for Topical Drug Delivery to Skin

The *stratum corneum* acts as a major barrier in delivering drugs to skin, especially for hydrophilic and larger drug molecules. The *stratum corneum* of skin consists of corneocytes and it is embedded in a lamellar structure. The unique arrangement of the corneocytes is the main reason for this layer to resist the permeation of molecules through it [68]. Skin temperature and hydration state are the two major factors that critically affect the permeation. Thermal analyses of skin have suggested that temperatures up to 70 °C do not significantly change the permeability coefficient, but temperatures above 70 °C cause irreversible denaturation and lead to drastic increase in the permeation coefficient. The *stratum corneum* maintains its hydration state of up to 80% relative humidity (RH), but an increase in hydration state due to humidity changes increases the diffusion coefficient. It has been observed that the penetration of lipophilic molecules increases with the increase in hydration of the *stratum corneum*. The chemical structure and physico-chemical characteristics of the drug molecule determine its permeability through the skin. Although the drugs with molecular weight of less than 500 Da are shown to have good permeation, there are a few exceptions, for instance heparin that has a molecular weight of 17,000 Da and still shows good penetration, which might be due to its inherent chemical nature [2].

### 3.3. Conventional Topical Delivery Systems in Treating Skin Diseases

The main objective of delivery drugs topically for skin diseases is to provide local and directed delivery to the diseased skin cells. The directed delivery of the drugs reduces the adverse effects that might otherwise occur when the drug is delivered systemically [1]. Treatment of chronic skin diseases such as psoriasis, atopic dermatitis and acne requires long-term use of topical medication. Topical drug delivery systems are the preferred delivery systems in such conditions and can be used in treating skin infections such as bacterial (impetigo, cellulitis), fungal (sporotrichosis, chronomycosis, blastomycosis), viral (herpes simplex virus, eczema) and autoimmune diseases (scleroderma, psoriasis) [69]. Conventional topical delivery systems suffer from several drawbacks such as repetitive dosing frequency, fluctuation in flux and so on, restricting them from being considered as effective topical products. Another drawback associated with conventional topical drug delivery systems is allergic reactions, which makes them less safe [68].

### 3.4. Nanotechnology-Driven Topical Drug Delivery Systems for Skin

Novel drug delivery systems are being studied to solve the various challenges linked with conventional systems, mainly through improvement of the penetration of drugs through the skin. The aim with the novel systems is to provide controlled drug release, which reduces the frequent application of medicament and reduces the undesirable side effects, as well as protecting the degradation of encapsulated drugs. Nanotechnology-driven drug delivery systems aid in site-specific skin targeting, which might result in greater drug retention at the target site [68]. The epithelial cells of skin have a negative charge on their surface due to which it is anticipated that the drug delivery systems carrying positive charge can easily interact with the cells, leading to increased permeability of the drug and a prolonged pharmacological effect [70]. Table 2 presents the nanotechnology-based topical formulation on the market or in clinical trials, and Table 3 summarizes the literature available on topical nano-based drug delivery systems for skin diseases.

### 3.5. Types of Nanotechnology-Driven Drug Delivery Systems Available for Delivering Drugs to Skin

#### 3.5.1. Liposomes

Liposomes can deliver drugs to the skin efficiently due to their lipid composition that is similar to epidermal cells of the skin, which helps them penetrate the skin barrier better than other dosage forms. They can localize the drugs at the site of action, reduce systemic absorption, and therefore minimize drug side effects [71]. Betamethasone 17-valerate (BMV) and diflucortolone valerate (DFV) loaded liposomes were formulated for treating atopic dermatitis (AD), which is a chronic inflammatory and relapsing skin disease. The liposomes showed 2.68- and 3.22-fold higher retention, respectively for BMV and DFV in *stratum corneum* and epidermis when compared with available commercial cream. Cell culture studies, trans-epidermal water loss (TEWL) measurements and histological observations confirmed that liposome in gel formulation was safe to be used in treating AD [72].

The elastic lipid-based vesicles (elastic liposomes) have recently gained interest in delivering drugs to skin. They are similar to conventional liposomes in morphology, but differ in function. They are highly deformable liposomes and are also called as transferosomes with better permeation through the skin barrier [73]. They are made of phospholipids and edge activators that destabilize the lipid bilayer of the vesicles by using skin penetrating peptides, such as trans-activating transcriptional activator (TAT), polyarginine or skin permeating and cell entering (SPACE)-peptide and result in deformability of the bilayer. This deforming ability of the edge activators affects the interfacial tension of the lipid bilayer [74]. Eline Desmet et al. designed an elastic liposome formulation for RNA interference (RNAi) to treat psoriasis. Cultured primary skin cells and psoriasis tissue model in vitro were used to prove the effectiveness of elastic liposomal carriers. Cationic liposomes deliver functional RNAi molecules to the different epidermal layers of normal or impaired skin, without crossing the dermal compartment. The psoriasis model was checked by down-regulation of hBD-2 and it was found that hBD-2 mRNA expression decreased to ~70% using cationic liposomes. From the finding it was suggested that these elastic liposomes are better carriers for RNAi delivery [73].

#### 3.5.2. Solid Lipid Nanoparticles

Solid lipid nanoparticles (SLN) are novel carriers that are composed of a solid lipid core coated with surfactant. The lipids used are non-irritating and non-toxic to the skin, and they are also considered safe to be applied on damaged skin. The small size of the lipid particles is shown to have a close interaction with *stratum corneum* and thereby the system increases the penetration, occlusion and accumulation of drug in the dermis region, which makes them the appropriate system for topical-targeted drug delivery. Pradhan et al. prepared fluocinolone acetonide SLN for treating psoriasis by emulsification-ultrasonication method. The authors prepared a gel-like formulation, which showed enhanced solubility of fluocinolone acetonide and helped the drug get deposited onto the epidermis, the location where psoriasis develops [75].

Irritant contact dermatitis (ICD), is one of the skin diseases manifested by edema, erythema, epidermal thickening, itching and scaling [76]. Presently, anti-histaminic, hydroquinones and topical corticosteroids are used for treating ICD, but long-term use of these drugs may lead to undesirable effects such as dry mouth, cataracts, high blood pressure, constipation, obesity, etc. Polyphenols are valuable compounds possessing scavenging properties towards radical oxygen species and complexing properties towards proteins. These abilities make polyphenols interesting for the treatment of various dermatological problems. Curcumin (CUR) is a naturally occurring polyphenol found in rhizomes of *Curcuma longa* Linn. CUR also exhibits antioxidant activity, which is beneficial for several skin-related problems such as eczema, dermatitis, pigmentation, acne, psoriasis lesions and all the exfoliative skin diseases. Due to the presence of high amounts of epidermal lipids within the *stratum corneum*, SLNs seem to be a promising delivery system as compared to other nanoparticulate carriers. Considering this, Shrotriya et al. designed curcumin-loaded SLN (CUR-SLNs) incorporated into carbopol gel. Occlusive test, irritation test on skin, tyrosine enzyme inhibition activity and antioxidant activity using commercial gel and SLN-loaded gel was performed on skin model. It was demonstrated that CUR-SLN gel showed efficient occlusion properties, and enhanced skin deposition, inhibition of tyrosinase enzyme activity and improved antioxidant activity compared to that of a conventional CUR-plain gel. ICD was developed using dinitrochlorobenzene (DNCB) on the dorsal surface of ears of mice. In vivo study suggested that SLN-loaded gels are superior in suppression of ear swelling and reduction in skin water content in ICD disease compared to commercial gel [76].

#### 3.5.3. Niosomes

Meng et al. formulated celastrol-loaded niosomal hydrogel to treat psoriasis, which increased the permeation of celastrol in the skin and thus increased its anti-psoriasis activity in a mice model [77]. Benzoyl peroxide (BPO) is the topical medication mostly used for the treatment of acne against the bacteria *Propionibacterium acnes*. BPO has poor aqueous solubility, forms cluster and it gets crystallized in aqueous environment. These clusters are not able to penetrate through the follicles which is why conventional formulations like cream, lotion and gel have higher concentration of the drug (2.5–10%). Goyal et al. encapsulated BPO in niosome gel and found that the formulation resulted in better permeation of the drug through the skin and also reduced skin irritation. It was also suggested that BPO-loaded niosomal gel causes better reduction in colony forming unit (CFU) count of *Propionibacterium acnes* bacteria in mice ear than plain BPO [78].

Topical application of 5-aminolevulinic acid (ALA) has been employed in photodynamic therapy (PDT) treatment of superficial skin carcinoma. According to the PDT protocol, a concentrated aqueous solution (20% *w*/*w*) of ALA is applied on the lesion, left for 3–6 h for drug absorption, and removed before the irradiation of light at the lesion. The main limitation of this therapy was the hydrophilicity of ALA due to which permeation of ALA through *stratum corneum* was hindered. ALA-loaded noisome gel was prepared to improve the permeation of the drug across the *stratum corneum*. Ex vivo skin permeation studies performed on excised human skin showed that the ALA-loaded niosomal formulation showed better penetration across the skin when compared to the aqueous solution of ALA [79]. El-Say et al. formulated a diacerein-loaded niosomal gel formulation that exhibited better anti-inflammatory activity compared to the commercial gel formulation. White albino rats were chosen as the animal model to evaluate anti-inflammatory activity and inflammation was developed by using the carrageenan raw paw method with slight modifications [80].

#### 3.5.4. Nanoparticles

Nanoparticles exhibit improved localized targeting of drug and thus are helpful in reducing systemic side effects. Mao et al. prepared CUR loaded polymeric nanoparticles to treat inflammation prevailing in psoriasis disease. The polymer used here was RRR-α-tocopherol succinate-grafted poly-lysine conjugate. Imiquimod (IMQ) induced psoriasis-like mouse model was used to test the prepared formulation. Tumor necrosis factor (TNF) is one of the most potent inflammation activators that play a major role in signaling the classical nuclear factor kappa B (NF-B). The contribution of NF-B may be significant in IMQ-induced psoriasis-like inflammation. The results showed that TNF-α, NF-B, IL-6 expression was increased in IMQ-treated skin, but after the treatment with CUR-NPs-gel/clobetasol their expression was significantly decreased. Angiogenesis inhibition study was performed using the prepared nanoformulation gel by quantifying cluster of differentiation 31 (CD31) expression visually after skin staining with immunofluorescent dye. CD31 are biomarkers that are highly expressed during new blood vessel formation. Large amounts of new blood vessel formation were found in the control group, based on the large numbers of immunoflouroscent CD31 that were seen visually in the controlled group. It was found that a much smaller quantity of CD was shown in CUR-NPs-gel. It was concluded that curcumin-loaded nanoparticle gel inhibited the inflammatory cascade triggered by TNF-α and the inflammation associated angiogenesis stimulated in psoriasis, improving antioxidant activity. Curcumin nanoparticles were formed using a novel cationic amphiphilic polymer, an RRR-α-tocopheryl succinate-grafted ε-polylysine conjugate (VES-g-ε-PLL). These cationic nanoparticles were incorporated into 8% silk fibroin through electrostatic interaction. The increase in skin permeation of curcumin was responsible for an improved response in psoriatic plaque-like model, suggesting a promising potential of this drug delivery system to treat inflammatory skin disorders like psoriasis [81].

**Figure 3:**
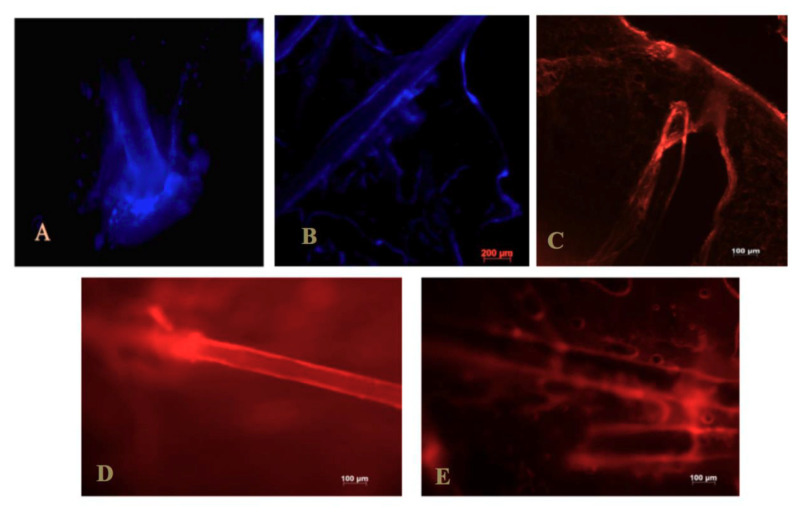
Fluorescence image of adapalene and Nile red localization within the hair follicles and epidermis. Fluorescent images taken from (**A**) the surface, (**B**) vertical section of porcine ear skin treated with adapalene-TyroSpheres, and (**C**) vertical section of porcine ear skin treated with Nile red-TyroSpheres. Cyanoacrylate surface biopsies of porcine ear skin treated with (**D**) Nile red-TyroSpheres, and (**E**) Nile red solution in propylene glycol. Reproduced with permission from [82], copyright 2017 Elsevier B.V.

Ramezanli et al. investigated adapalene (drug for the topical treatment of acne) loaded tyrosine-derived polymeric nanoparticles (also known as TyroSpheres). The formulation was evaluated ex vivo using human cadaver and porcine ear skin and compared with the marketed adapalene formulation (Differin^®^). It was reported that the crystallinity of adapalene was decreased and the TyroSpheres got accumulated in the hair follicles, due to which the topical delivery of adapalene was improved as compared to the commercial product. Drug distribution in the hair follicles was visualized using adapalene inherent fluorescence and Nile red loaded TyroSpheres with the aid of a fluorescence microscope (Figure 3). Improved follicular delivery of the TyroSpheres was attributed to the small particle size [82].

Balzus et al. formulated dexamethasone-loaded Eudragit RS and ethyl cellulose nanoparticles, which adhere well to the skin and penetrate through the hair follicles. The nanoparticles released the dexamethasone drug in a controlled fashion compared to the commercially available cream in ex vivo studies. [83].

#### 3.5.5. Polymeric Micelles

Polymeric micelles are capable of disrupting the *stratum corneum* layer, the major barrier for delivering drugs to skin, hence facilitating entry of drugs through skin [84]. Abd-elsalam et al. prepared polymeric mixed micelles containing Cremophor EL as penetration enhancer. It was observed that the mixed micelle formulation of terconazole with Cremophor EL remained stable for three months at room temperature. To know the amount of drug deposited in the skin, ex vivo studies were performed using rat skin. Micelles containing Cremophor EL showed higher permeation as compared to the drug suspension and mixed micelle lacking Cremophor. It was suggested that the addition of Cremophor EL had intensified the interaction of the prepared micelles with keratin in the corneocytes. The in vivo histo-pathological study showed no inflammation or skin irritation, thus suggesting the safety of prepared micelles for topical application [84].

Vismodegib is a drug initially approved for the treatment of metastatic or basal cell carcinoma, the most prevalent human skin cancer. On systemic administration, vismodegib causes serious side effects like gastrointestinal, musculoskeletal and connective tissue disorders, which sometime lead to death. Hence, Kandekar et al. developed vismodegib-loaded mPEG-hex PLA polymeric micelles and formulated them as gel using hydroxyl ethyl cellulose. Their findings suggests that the polymeric micelles caused an 8000-fold increase of the aqueous solubility of vismodegib and enabled the delivery of supra-therapeutic amounts of the drug to the basal epidermis and deeper tissue [85].

Vitamin D3 is known to have anti-psoriatic activity, but it is highly lipophilic and sensitive to moisture, heat and light, which can adversely affect its bioactivity. Ramezanli et al. prepared vitamin D3-loaded TyroSpheres (polymeric micelles) for topical skin delivery to treat psoriasis. The skin distribution studies showed that vitamin D3-TyroSpheres with 15 wt% loading were efficient in delivering the drug to the epidermis and dermis. It was also observed that the exposure time of skin with to micellar formulation (from 6 to 12 h) increased vitamin D3 diffusion significantly to the epidermis. On the other hand, increasing the exposure time of commercially available vitamin D3 Transcutol^®^ did not show any increase in deposition of drug to epidermis [86].

Fabrication of IMQ-loaded nanosized (d_n_ = 27 nm) polymeric micelles was reported using block co-polymer methoxyPEG-hexyl-substituted PLA (mPEG-hexPLA) for the treatment of skin cancer. IMQ micelles (0.05%) were then incorporated in gel using carboxy methyl cellulose sodium (CMC) and tested on porcine and human skin to evaluate their efficiency. It was found that prepared gel is more efficient in delivering IMQ in the different layers as compared with the Aldara™ cream [87].

## 4. Wounds and the Barriers for Topical Drug Delivery to Wounds

Wounds can be classified based on the nature of healing, thickness and microbial load. Acute or chronic wounds are based on the nature of healing. In acute wounds, the wound healing time is 8–12 weeks. In chronic wounds, anatomical and functional integrity of the skin is not restored for a period of over three months. Chronic wounds include pressure ulcers, vascular ulcers and diabetic foot ulcers [103]. Based on the thickness of the wound, they can be classified as superficial, partial and full thickness wounds and can be further classified as clean, clean-contaminated, contaminated and infected wounds based on their microbial load. Nussbaum et al. conducted a retrospective analysis of the Medicare 5% Limited Data Set to determine the cost of chronic wound care for Medicare beneficiaries. The study concluded that the Medicare cost for surgical wounds and infected diabetic foot ulcers was 38.3 billion and 18.7 billion US dollars respectively [104].

Endogenous factors are used for wound healing clinically, but their clinical use is limited due to their breakdown by the proteolytic enzymes that are present at the wound site. The wound environment has various pro-inflammatory cytokines that can deactivate drugs. Topical use of growth factors for wound healing is limited because of the very short half-life of the proteins due to the close control and inactivation by protease inhibitors. Blood flow is poor at the site of wound healing. Therefore, systemic drug delivery is also not of significant importance in wound healing. The dysfunctional wound bed vasculature reduces the bioavailability of compounds that are administered orally or intravenously. Hence, the wound environment requires complex drug delivery systems that can deliver the drugs and growth factors to the appropriate site [105].

### 4.1. The Process of Wound Healing

**Figure 4:**
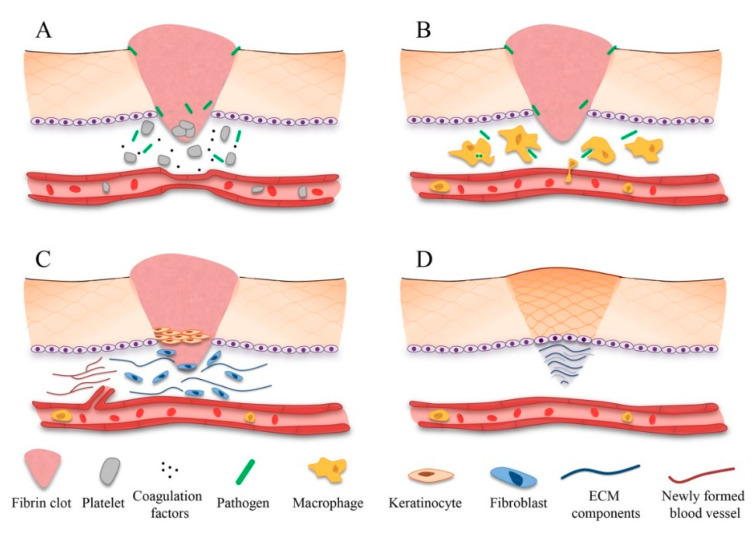
Wound healing process: (**A**) hemostasis, (**B**) inflammation, (**C**) proliferative phase and (**D**) remodeling phase. Reproduced with permission from [106], 2017, copyright 2017 Elsevier B.V.

Wound healing is a complex event that is designed to restore the normal functions of the skin and reduce the risk of infection and further complications [103]. Many factors are associated with poor wound healing, but the major role has been played by an abnormal and consistent inflammatory response, which leads to an excessive proteolytic activity due to the proteolytic enzyme degradation resulting in inflammation-mediated tissue damage. Decreased blood supply to the wound leads to ulcer formation. There are four phases in wound healing, namely, hemostasis, inflammation, proliferation and remodeling (Figure 4) [106].

Homeostasis involves platelet aggregation, immunity activation, blood clotting and complements system induction. Inflammation includes the recruitment of neutrophils and macrophages with extra cellular matrix (ECM) molecule production by resident cells in the skin. Proliferation involves re-epithelialization, angiogenesis and granulation. The expansion of keratinocytes epithelial cells, stem cells and fibroblasts is known as re-epithelialization. Activation of endothelial cells and the creation of a site in which these cells can proliferate in the wound is called angiogenesis. Granulation is defined as the promotion of tissue granulation by fibroblasts, granulocytes and macrophages. Finally, remodeling involves the production of collagen- and elastin-including fibroblasts leading to myofibroblasts in the presence of T cells and macrophages [106]. Monocyte chemoattractant protein 1, cytokines (interlukin-1 and interlukin-6), growth factors such as fibroblast growth factor (FGF), epidermal growth factor (EGF), transforming growth factor-β (TGF-β), platelet-derived growth factor (PDGF), bone morphogenic proteins (BMPs), connective tissue growth factor (CTGF), granulocyte macrophage-colony stimulating factor (GM-CSF) and vascular endothelial growth factor (VEGF) participate in wound healing.

### 4.2. Role of Nanotechnology in Wound Healing

Numerous strategies based on nanotechnology are available for wound healing and are summarized in Table 4.

#### 4.2.1. Nanoparticles

It has been reported that deficiency of epidermal growth factor (EGF) is one of the causes of diabetic foot ulcer (DFU). The effective concentration of exogenous recombinant human EGF (rhEGF) in wound for the treatment of DFU could not be achieved after local administration due to the short biological half-life of rhEGF, rapid dilution by tissue fluid, leakage from the wound surface, and degradation by enzymes. In order to overcome these challenges, Chu et al. designed rhEGF nanoparticles using PLGA polymer. These nanoparticles showed a controlled release of rhEGF for up to 24 h. These nanoparticles possessed better wound healing effects than those of pristine rhEGF [107]. LL37, an anti-microbial peptide/host defense peptide, which is part of the innate immune system, is known to promote wound healing. Chereddy et al. prepared LL37 antimicrobial peptide-loaded PLGA nanospheres and found that compared to LL37 alone, PLGA-LL37 nanoparticles significantly improved wound healing activity. The healing effect of PLGA-LL37 NP included higher re-epithelialization, granulation, tissue formation and immunomodulation [108].

Inorganic nanoparticles, especially metal oxide nanoparticles (MONPs), are also studied for the speedy recovery of wounds. MONPs like zinc oxide (ZnO), titanium dioxide (TiO_2_) and silicon dioxide (SiO_2_) play important roles in the production of reactive oxygen species, which stimulate the proliferation of fibroblasts and wound healing [109].

**Figure 5:**
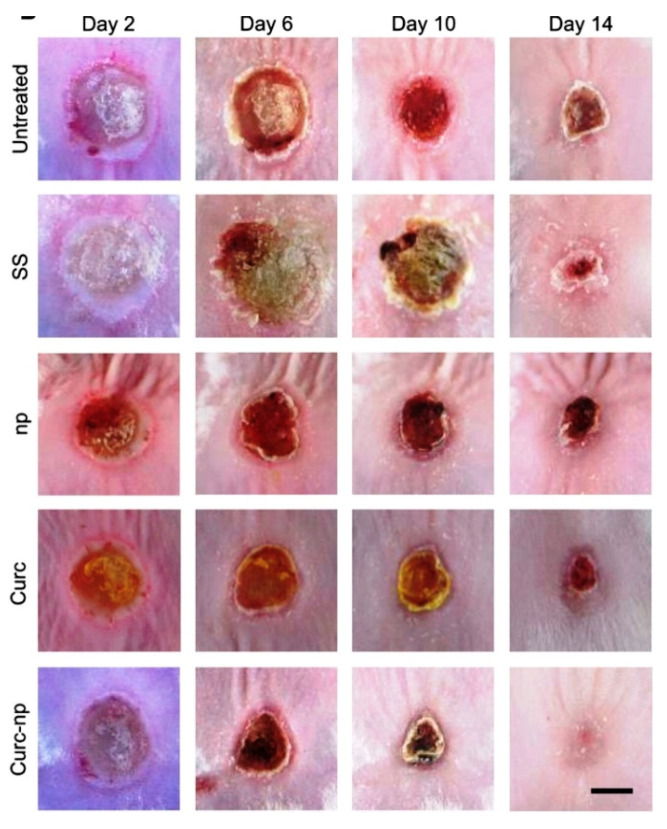
Hotographs of the wound healing process in a murine burn model treated with curcumin nanoparticles (curc-np) compared to untreated, silver sulfadiazine (SS), control np (np) and curcumin (curc). Scale bar = 5 mm. Reproduced with permission from [111], copyright 2015 Elsevier B.V.

In a study, *Origanum vulgare* (as aqueous leaf extract) engineered with titanium dioxide nanoparticles was applied topically on the wound for improving the therapeutic efficacy of the extract. The results suggested significant wound healing activity in Albino rats, established by measuring wound closure, histopathology and protein expression profiling [110]. Bacterial infection is often a major complication associated with burn wounds. Curcumin is a naturally derived substance with innate antimicrobial and wound healing properties, but since it has poor aqueous solubility and rapid degradation, encapsulating it within a protective carrier is necessary. Krausz et al. encapsulated curcumin in chitosan nanoparticles (size—222 ± 14 nm) using sol-gel-based formulation for wound healing purposes in the setting of skin infection. This novel topical gel formulation showed significantly reduced bacterial load and improved wound healing in murine burn model compared to other groups (Figure 5). Histological analysis showed increased granulation tissue formation, improved collagen proliferation and enhanced neovascularization [111].

#### 4.2.2. Solid Lipid Nanoparticles and Nanostructured Lipid Carriers

Solid lipid nanoparticles (SLN) and nanostructured lipid carriers (NLC) are efficient and non-toxic drug carriers, which can provide high drug concentration at the site of wound. SLNs consist of solid lipids or blending of solid lipids whereas NLCs are a mixture of solid lipid with liquid lipids. In NLC, imperfection is more as compared to the SLN, hence, it avoids expulsion of drugs from the matrix as compared to the SLNs which have fewer imperfections due to their high order structure. There is close contact between these nanoparticles and the skin due to their small size and lipid content. NLCs are very suitable for dermal administration because they can remain on the skin and release drugs in a sustained manner and are also known to improve skin hydration [103]. Many growth factors (GF) have been identified as mediators in wound healing. Hence, applying GFs exogenously is expected to repair the damaged tissue and accelerate the wound healing mechanism.

Gainza et al. encapsulated recombinant human epidermal growth factor (rhEGF) into SLN and NLC. The in vitro evaluation involved determining the bioactivity of rhEGF using fibroblasts and keratinocytes. The in vivo experiments concluded that 10 and 20 μg of rhEGF-loaded SLN and NLC were more effective in the wound healing process compared with the same number of intralesional doses of 75 μg of free rhEGF nanoparticles, and as effective as a single dose of 75 μg MS-rhEGF in genetically diabetic db/db mice (diabetic) [112].

#### 4.2.3. Liposomes

Stromal cell-derived factor-1 (SDF-1) is a chemokine known to increase the rate of healing of excisional skin wounds in mice. Olekson et al. formulated a cellular dermal scaffold loaded with SDF-1 liposomes and evaluated in a diabetic mouse excisional wound model with free SDF-1. The SDF-1 liposomes maintained and promoted sustained proliferation of SDF-1 in the wound that led to a positive effect on wound closure [113]. Since the peripheral perfusion is limited and wound healing is retarded in diabetic patients, such conditions needs special care. Therefore, Fukui et al. prepared liposome-encapsulated hemoglobin with high O_2_ affinity to increase tissue perfusion, suppress inflammatory cytokines and promote faster skin wound healing. The results suggest that the above-mentioned parameters were significantly improved in diabetic dB/dB mice and are comparable to normal mice when treated with liposomal formulation [114].

#### 4.2.4. Nano Implants

Implants placed in any soft tissue induce a cell-mediated inflammatory response immediately following implantation. The inflammatory response at the site of the wound involves inflammatory cells, reactive oxygen species, and nitrogen oxygen (NO) species; TiO_2_ is believed to have the ability to inhibit these reactive oxygen species. Smith et al. modified a titanium implant surface via TiO_2_ nanotubes to increase the TiO_2_ surface area and eventually the catalytic degradation of NO species. It was observed that the TiO_2_ nanotubes reduced the levels of the pro-inflammatory signaling molecule NO compared with an implant having a planar TiO_2_ surface [121]. Similarly, a coating of sandblasted and acid-etched surface dental implants with hydroxyapatite nanoparticles to increase the healing process [122]. Nanosurface-modified implants significantly increase the bone/implant contact and thus improve the osseointegration and neovascularization process and ultimately accelerate the healing process [123].

## 5. Toxicological Aspects of Topically Applied Nanoformulations

Toxicity is a prime concern for any topical formulation and the establishment of a toxicity profile is a mandatory requirement for regulatory approval. Nanoparticles have the potential to penetrate the cells and can produce free radicals, which leads to oxidative stress and collagen depletion. This depletion induces keratinization, atrophy of the dermis and skin wrinkling [124,125]. In one research finding it was found that keratinocyte proliferation was significantly inhibited after exposure of nanocrystals-coated dressing [126].

The various critical quality attributes including the polymer concentration, drug characteristic and critical process parameters of the topical formulation can lead to remarkable differences in terms of stability, safety, efficacy and toxicity. In a study, the authors investigated the signs of toxicity after exposure of *o*/*w* formulation comprising 5% hydroquinone (HQ) and a different base. The major challenge associated with HQ is its poor permeability and physicochemical stability, which leads to the use of p-benzoquinone (pBQ) in the formulation which is known as carcinogenic [127,128,129].

The high HQ concentration was found in skin from formulation 1 containing Beeler’s based plus antioxidants, followed by formulation 2, which contains Beeler’s base and dimethyl isosorbide as solubilizer. This interaction might be between the cationic proteins of the skin and the anionic nature of Beeler’s base (sodium lauryl sulfate). Some signs of toxicity were also observed on the skin with all topical semisolid *o*/*w* formulation with 5% HQ after 6 h of exposure [130].Wu and co-workers reported the dermal exposure of titanium dioxide (TiO_2_) nanoparticles in vitro and in vivo using isolated porcine skin. The results suggest that the pathological lesions are likely to occur probably due to oxidative stress induced by the deposition of TiO_2_ nanoparticles. They also reported that with a prolonged exposure time, this topically applied nano-TiO_2_ can induce skin aging and may pose health risk issues to humans after long dermal exposure [131].

Trop and co-workers prepared silver nanoparticles for the treatment of wound healing. Silver coated dressing acticoat caused liver enzymes and argyria-like symptoms in acute burn patients (30% burns). The silver levels in urine and plasma were monitored regularly to avoid the toxic effect of silver in burn patients. After 1 week of local treatment, hepatotoxicity and argyria-like symptoms and grayish discoloration of the patient’s face was also reported [132]. Numerous reports are available mentioning the advantages of nanotechnology-based dermal formulations but their toxicity assessments are scarce, albeit its notable significance in clinical translation.

## 6. Conclusions

Conventional drug delivery systems for topical delivery possess several challenges, including repetitive dosing administration and rapid release, which impact product performance in healthcare sectors. Nowadays, nanotechnology-driven topical complex formulations such as polymeric micelles, liposomes, niosomes, nanoparticles, and implantable devices are gaining significant attention in the pharmaceutical sector for topical applications.

Nanotechnology has provided minimally invasive ocular drug delivery systems. The invasive nature of intravitreal and periocular injection has spurred researchers to work on topical delivery systems that offer higher patient compliance. The nanotechnology-driven topical drug delivery systems to eyes possess properties such as enhanced residence time in the pre-corneal area, better permeation ability through the ocular barriers, sustained delivery of drugs at targeted sites, ultimately improving therapeutic efficacy. Although the in vivo studies have shown that the nanotechnology-based drug delivery systems did not produce any irritation and inflammatory response, their long-term effect must be properly analyzed. The existing topical formulations to treat skin diseases face challenges including a smaller amount of drug penetrating the skin barrier, systemic absorption leading to unwanted side effects and, if administered orally, adverse effects and poorly targeted therapy. Psoriasis, skin cancer, fungal infections and bacterial infections are a few of the most common skin diseases that require topical treatment. Most of the in vivo studies performed have shown better skin permeability of the nanoformulations, their ability to reach the diseased site, being free from irritation and inflammatory responses. However, extensive investigation has to be carried out to translate the novel formulations for clinical use. Nano-based systems certainly exemplify an improvement in safety and efficacy for topical drug delivery and hold great potential for efficient treatment leading to improved quality of the patient’s life.

## 7. Future Prospects

Nanotechnology is still a promising and emerging field, presently focusing on encapsulating drugs which require a low dose for treatment. Several products encompass technical challenges, e.g., (i) nanoparticles have a low loading capacity, (ii) dendrimers show toxicity problems, and (iii) liposomes have stability challenges. Scale-up, batch-to-batch reproducibility, stability and clinical performance can be regarded as the major challenges in the clinical translation from industry perspectives. The number of nanotechnology-based products going into the market is smaller compared to the efforts that have been put at the research level. We need to focus on the technology transfer, scale-up and validation of these products in R&D laboratories for clinical translation. Amalgamation of nanotechnology with other delivery systems such as implants, nanofibers, hydrogels, etc., may be further investigated for improved results in drug delivery by topical route. The main challenges that researchers need to focus on for clinical translation are retention time, vision interferences, nano-bio interactions, drug permeation and accumulation.

## Figures and Tables

**Table 1:** Various nanoformulations used for ocular delivery (nonclinical study).

S. No	Formulations	Drug	Ailment	In Vivo/Ex Vivo Study	Inference	Reference
**1.**	Liposomes	Voriconazole	Fungal keratitis	Ex vivo static permeation through porcine cornea	Voriconazole liposomes made of soyphosphatidylcholine were successful in delivering drug through the cornea for treating fungal keratitis with no irritation in eyes reported.	[38]
**2.**	Annexin A5-associated liposomes	Bevacizumab	Posterior segment ocular diseases such as glaucoma or neovascular age-related macular degeneration (AMD)	In vivo assays performed in rat eye and rabbit retina	Annexin A5 (AnxA5) is a calcium-dependent phospholipid binding protein whose addition to phospholipid vesicles(PLVs) was found to significantly increase the concentration of encapsulated Avastin (bevacizumab) reaching the posterior segment of the rat eye when compared to an equivalent concentration of PLVs in the absence of AnxA5, or a higher concentration.	[58]
**3.**	Dendrimers	Dexamethasone	Diabetic retinopathy	In vivo ocular distribution study in Sprague-Dawley rats	Topical delivery of dexamethasone-PAMAM dendrimers increased ocular bioavailability and resulted in increased concentration of the drug in retina.	[51]
**4.**	Dendrimer hydrogel	Brimonidine and timolol maleate	Glaucoma	Ex vivo studies in freshly excised bovine eyes	Brimonidine and timolol maleate were encapsulated into dendrimers hydrogel (DH) and it was found that the transport of the drugs across the bovine corneal endothelium was significantly increased as compared to the eye drop solution.	[50]
**5.**	Polymeric nanoparticles	Amikacin	*Staphylococcus aureus* infection	In vivo studies male New Zealand albino rabbits	The polymeric nanoparticles prepared using nano emulsification method showed controlled release of the drug as compared to the commercial eye drop. The formulation was stable and did not show any irritation on application.	[59]
**6.**	Galactosylated chitosan nanoparticles	Timolol maleate	Glaucoma	In vivo pharmacodynamic studies in New Zealand albino rabbits	This formulation improved the drug permeation through cornea. The in vivo pharmacodynamic study showed that the formulation substantially improved the drug efficacy and improved its bioavailability.	[60]
**7.**	Chitosan/PLA nanoparticles	Rapamycin	Immunosuppression in corneal transplantation	In vivo studies in New Zealand rabbits	Chitosan/PLA nanoparticles showed better retention properties at the precorneal site as compared with rapamycin aqueous suspension. The nanoparticles showed an excellent immunosuppressive effect compared with the rapamycin eye drops.	[61]
**8.**	Eudragit RS100 and RL100 polymeric nanoparticles	Gatifloxacin	Ocular infections such as conjunctivitis, keratitis, and endophthalmitis	Tested on bacteria	The Gatifloxacin-loaded nanoparticles were shown to improve bioavailability by prolonging the retention of the drug in the eyes.	[41]
**9.**	Solid lipid nanoparticles	Itraconazole	Fungal corneal infections	In vitro studies with goat cornea	Itraconazole solid lipid nanoparticles were prepared using stearic acid and palmitic acid. It was concluded that permeation of itraconazole from stearic acid SLNs was higher than palmitic acid SLNs. The formulation showed better antimicrobial efficacy.	[62]
**10.**	Gellan gum polymeric nanoparticles	Doxycycline	Corneal neovascularization, recurrent epithelial erosions and sterile corneal ulcerations	Eye irritancy test in male New Zealand albino rabbits	Doxycycline nanoparticles showed sustained release of the drug with no irritant properties. Antibacterial studies showed that the formulation inhibited bacterial growth at very low concentrations than that of the pure drug.	[63]
**11.**	Chitosan nanoparticles	Ketorolac tromethamine	Post-operative eye inflammation	Ex vivo permeation studies with cornea obtained from porcine eye balls	The in vitro release study performed suggested that the prepared formulation is capable of sustaining drug release over a period of 6 h as compared to ketorolac tromethamine solution that releases the drug rapidly over a period of 3 h.	[43]
**12.**	Nanomicelles	Pimecrolimus	Keratoconjunctivitis Sicca	In vivo test in Kunming (KM) mice	The formulation resulted in higher drug encapsulation capability with drug loading and encapsulation efficiency of 7.57% ± 0.10% and 97.9% ± 1.26%, respectively. It was found that the nanocarrier protects the eyes from drug-induced toxicity and vision loss. The prepared nanomicelar formulation inhibits the cytokine production and shows a significantly increased healing process as compared to the other group.	[64]
**13.**	Niosome	Gentamicin	Ocular infections	In vivo Ocular irritancy test performed on albino rabbits	The in vitro evaluation of gentamicin niosomes showed that the niosomes made of tween 60, cholesterol and diacetyl phosphate prolonged the release of the drug as compared to the gentamicin solution.	[40]
**14.**	PLGA nanoparticles	Loteprednol etabonate	Ocular inflammation	Ex vivo transcorneal permeation profile of optimized PLGA nanoparticle formulation was assessed on excised goat cornea	The prepared formulation showed better penetration of the drug across excised goat cornea and adhered to the ocular surface for a prolonged period as compared to the pure drug suspension.	[65]

**Table 2:** Nanotechnology-driven products in clinical studies and market for topical applications.

S. No.	Product	Drug	Formulation	Application	Current Status	References
**1.**	Dermos™	Paclitaxel	Nanosomal formulation with diameter less than 1 nm	AIDS-associated Kaposi’s sarcoma	On the market	[66]
**2.**	Estrasorb	Estradiol hemihydrate	Micellar nanoparticles, an emulsion of estrogen and soybean oil	Prevention of hot flushes and treatment of vasomotor symptoms associated with menopause	On the market (FDA approval)	[88]
**3.**	Pevaryllipogel	Econazole	Liposomes	Anti-fungal	On the market	[89]
**4.**	NB-001	--	NanoStat™ topical formulation technique for specifically targeting microbes	Treatment of cold sores associated with herpes labialis	Clinical trial	[66]

**Table 3:** Reported topical nanoformulations used in the treatment of various skin conditions.

S. no.	Nano Formulation	Drug	Ailment/Disease	In-Vivo/Ex-Vivo Model	Results	References
**1.**	Solid lipid nanoparticles	Tretinoin	Psoriasis, acne, photoaging and epithelial skin cancer	Ex vivo permeation and irritation study using whister rats.	Prepared formulation improved photo stability and toleratability, reduced irritation, and increased drug permeation as compared to the free drug.	[90]
**2.**	Polymeric nanoparticles (Lecithin-Chitosan)	Clobetasol-17-propionate	Inflammatory skin diseases	In vitro permeation study through franz diffusion cell.	Polymeric nanoparticles reduced side effects of the drug as compared to the marketed creams. Prepared nanoparticles increased epidermal targeting.	[91]
**3.**	Nano emulsion-based gel	Clobitasol propionate and calcipotriol	Psoriasis	Ex vivo permeation study is performed using pig ear skin. In vivo efficacy was performed using BALB/c model.	Optimized formulation showed higher anti-psoriatic activity as compared to the free drug.	[92]
**4.**	Nanoethogel and nanogel formulations	Amphotericin B	Dermatophytes and surface fungal infections	Ex vivo permeation study through rat skin and porcine ear skin	It was found that Strat-M™ is a better alternative to carry out skin permeation experiments due to the consistent results, reproducibility, easy availability.	[93]
**5.**	Nanogel	Spantide II and ketoprofen	Allergic contact dermatitis and psoriasis	Ex vivo permeation study was performed using human skin psoriatic plaque like model was developed on C57BL/6 mice	Deposition of drugs was increased 8.5- and 9.5-fold in dermis and epidermis, respectively, as compared to the free drug. Prepared formulation deposited drugs in deeper tissues.	[94]
**6.**	Chitosan–tripolyphosphate nanoparticles	Aciclovir	Herpes infections	In vitro permeation studies with porcine abdominal skin	Incorporation of aciclovir into chitosan-tripolyphosphate nanoparticles significantly improves its chemical stability. Nanoparticle formulation improved permeation as compared to the free drug.	[95]
**7.**	Nano emulsion-gel	5-Fluorouracil	Actinic keratosis and Non-melanoma skin cancers	Ex vivo permeation study using rat, goat and cow skin.	It was found that prepared nanoemulsion gel increased permeation by 1.2 fold in rat skin and 12.51 in the goat skin. Prepared formulation was safer compared to the free drug.	[96]
**8.**	Solid lipid nanoparticle and Nanostructure lipid carrier	Tacrolimus	Psoriasis	Ex vivo permeation study through pig ear skin Anti-psoriasis was model developed using mice model	Tac liquid crystal nanoparticle (LCNP) Tac-SLN, Tac-NLC and Tac-liposome-loaded gels showed 14-, 11.5-, 12.5- and 3.7-fold increments in dermal bioavailability respectively, in comparison to free Tac-loaded gel.	[97]
**9.**	Nanoemulgel	Aceclofenac and capsaicin	Psoriasis	Ex vivo permeation study was performed using human skin	Nanoemulgel showed a controlled release drug pattern as compared to free drug. It was also found that prepared formulation showed 2.02- and 1.97-fold higher permeation as compared to their respective free drugs.	[98]
**10**	Solid lipid particles and nano emulsion	Triptolide	Anti-inflammatory	carrageenan-induced inflamation model was developed using wistar rats	Improved availability of drug at target size, reduced side effect like irritation and staining.	[99]
**11**	Nanostructured lipid carrier	Betamethasone	Atopic dermatitis	Ex vivo permeation study was performed using rabbit skin	Drug-loaded lipid carrier showed high retention as compared to the free drugs.	[100]
**12**	Nanoparticle delivery	Dacarbazine	Melanoma	In vitro permeation study through franz diffusion cell	Rate of drug release was higher in nanoparticles as compared to the suspension of the drug.	[101]
**13**	Niosomes	Methotrexate	Psoriasis	In vivo skin deposition study using wistar rats	Results showed that targeted MTX delivery might be achieved using topically applied niosomes for enhanced treatment of psoriasis.	[102]

**Table 4:** Reported nanoformulations for wound healing.

S. No.	Nano Formulation	Drug	Inferences	Reference
**1.**	Methoxy poly(ethylene glycol)-graft-chitosan (-mPEG) film	Curcumin	Applied in full-thickness punch wounds model of SD (Sprague dawley) rats showed faster wound reduction and shortened re-epithelialization period as compared to the MPEG-chitosan film. Masson’s trichrome staining indicated that the wound treated with curcumin-MPEG-chitosan film had a compact and well-aligned collagen as compared MPEG-chitosan film treated wound.	[115]
**2.**	Poly (lactic-co-glycolic acid) nanoparticles	Ferulic acid (FA)	In vivo studies showed that FA nanoparticles applied topically hydrogel and administrated orally (dispersion) promoted wound healing in diabetic rats.	[116]
**3.**	Nanoparticles incorporated in hydrogel	Zinc oxide	ZnO causes toxicity to the fibroblast cells at higher concentration. Hydrogel containing 4.98% of ZnO nanoparticles showed complete monolayer formation.	[117]
**4.**	Liposomes prepared using DSPC and DSPA and cholesterol	Stromal cell-derived factor-1 (SDF-1)	The SDF-1 liposomes maintained and promoted sustained proliferation of SDF-1 in the wound that led to positive effect on wound closure.	[113]
**5.**	Soluplus nanodispersion	Proanthocyanidins	The formulation showed antibacterial activity against *E. coli*, *S. aureus* and *Bacillus*. The bacterial cell membrane became more permeable and the cell structure was disrupted. It was found that this formulation could improve wound healing without forming scars. The histopathological assay of the treated animals showed complete re-epithelialisation, migration of cells, proliferation of cells and fibroblast attachment.	[118]
**6.**	Gold nanoparticles	Gold	Histological examinations showed that gold nanoparticles in photo biomodulation therapy were found to be more effective in contracting and accelerating wound healing due to enhanced epithelialization, collagen deposition and fast vascularization.	[119]
**7.**	Chitosan PEG and tetramethyl orthosilicate nanoparticles	Curcumin	The formulation can be used to treat the burn wounds efficiently reducing bacterial load and enhancing wound healing.	[111]
**8.**	Solid lipid nanoparticles (5% (*w*/*v*) Precirol® 133 ATO 5) and nanolipid carrier (Precirol® 146 ATO 5 and 20 mg of Miglyol® 182) (SLN and NLC)	Recombinant human epidermal growth factor (rhEGF)	The bioactivity of the solid lipid nanoparticles was higher in the cell lines studied as compared to the free rhEGF.	[112]
**9.**	PLGA nanoparticles	Vascular endothelial growth factor (VEGF)	The VEGF released from the PLGA-VEGF nanoparticles induced neovascularization significantly and did not show any cytotoxicity. It was suggested that the formulation accelerated wound closure by targeting different cells involved in wound healing.	[120]
